# Gut-bacteria derived membrane vesicles and host metabolic health: a narrative review

**DOI:** 10.1080/19490976.2024.2359515

**Published:** 2024-05-29

**Authors:** Jari Verbunt, Johan Jocken, Ellen Blaak, Paul Savelkoul, Frank Stassen

**Affiliations:** a Department of Medical Microbiology, Infectious Diseases & Infection Prevention, School of Nutrition and Translational Research in Metabolism (NUTRIM), Maastricht University Medical Center+, Maastricht, The Netherlands; b Department of Human Biology, School of Nutrition and Translational Research in Metabolism (NUTRIM), Maastricht University Medical Center+, Maastricht, The Netherlands; c Department of Medical Microbiology and Infection Control, Amsterdam University Medical Centers, Amsterdam, The Netherlands

**Keywords:** Microbiota, metabolic disease, bacterial membrane vesicles, obesity, insulin resistance

## Abstract

The intestinal microbiota, consisting of an estimated 10^10–10^11 organisms, regulate physiological processes involved in digestion, metabolism, and immunity. Surprisingly, these intestinal microorganisms have been found to influence tissues that are not directly in contact with the gut, such as adipose tissue, the liver, skeletal muscle, and the brain. This interaction takes place even when intestinal barrier function is uncompromised. An increasing body of evidence suggests that bacterial membrane vesicles (bMVs), in addition to bacterial metabolites such as short-chain fatty acids, are able to mediate effects of the microbiota on these host tissues. The ability of bMVs to dissipate from the intestinal lumen into systemic circulation hereby facilitates the transport and presentation of bacterial components and metabolites to host organs. Importantly, there are indications that the interaction between bMVs and tissues or immune cells may play a role in the etiology of (chronic metabolic) disease. For example, the gut-derived bMV-mediated induction of insulin resistance in skeletal muscle cells and pro-inflammatory signaling by adipocytes possibly underlies diseases such as type 2 diabetes and obesity. Here, we review the current knowledge on bMVs in the microbiota’s effects on host energy/substrate metabolism with a focus on etiological roles in the onset and progression of metabolic disease. We furthermore illustrate that vesicle production by bacterial microbiota could potentially be modulated through lifestyle intervention to improve host metabolism.

## Introduction

One of the greatest health challenges of the 21st century is the increase in occurrence of obesity and obesity-related metabolic disorders including metabolic syndrome (MetS) and type 2 diabetes (T2D).^1^ As of 2019, more than 2 billion people globally are overweight (Body Mass Index (BMI) ≥ 25 kg/m^2^), and approximately 800 million of these are obese (BMI ≥ 30 kg/m^2^).^2^ In MetS and obesity, whole-body energy and substrate metabolism is altered, thereby increasing the risk of cardiovascular disease, diabetes, cancer, and all-cause mortality.^3^ Obesity is often associated with insulin resistance and low-grade inflammation of insulin-sensitive tissues including adipose tissue (AT), skeletal muscle, and the liver.^4,5^ A concomitant lipid overflow into the circulation and consequently, lipid storage in non-ATs contributes to the onset and progression of insulin resistance and aberrant glucose metabolism in skeletal muscle and the liver.^6^

**Figure 1.**
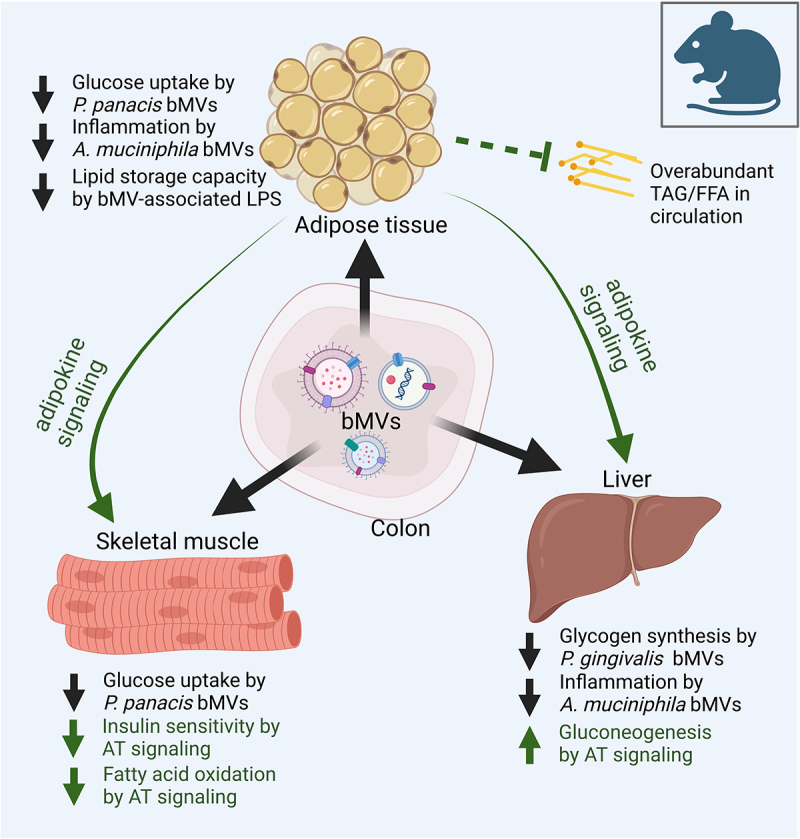
A central role for bacterial membrane vesicles in glucose metabolism and metabolic disease. Illustrating reported effects of gut-derived bMVs on adipose tissue (AT), skeletal muscle tissue and the liver in metabolic disease, with a secondary focus on the consequential signalling effects of AT on skeletal muscle cells and the liver. In mice, gut-derived *Pseudomonas panacis* bMVs decreased glucose uptake in AT and skeletal muscle^23^ whereas the translocation of *Porphyromonas gingivalis* bMVs to the liver reduced glycogen synthesis in response to insulin signalling.^25^ Interestingly, oral administration of pasteurized *Akkermansia municiphila* and its bMVs decreased HFD induced measures of inflammation (Interleukin 6 (IL6), Tumor Necrosis Factor alpha (TNFα)) in AT and the liver in mice.^24^ As master regulator of insulin and glucose homeostasis, AT removes FFAs and TAGs from systemic circulation, thereby preventing lipotoxicity induced insulin resistance in liver and skeletal muscle tissue.^6^ Via endocrine signalling AT delivers cytokines and adipokines to skeletal muscle tissue and the liver, decreasing insulin sensitivity and fatty acid oxidation in the first, and increasing gluconeogenesis in the latter.^88^ For the sake of simplicity, the effects of the interaction between the microbiome, its vesicle produce and the cells lining the intestinal barrier on metabolic health, as well as the role of tissue-infiltrating immune cells and crosstalk between skeletal muscle cells, liver and other organs are not illustrated here. TAG: triacylglycerol. FFA: free fatty acids.

Importantly, the onset and progression of obesity and MetS has often been found concerted with gut microbial dysbiosis, in which the composition and/or functioning of the microbes inhabiting the gastrointestinal tract is imbalanced.^7,8^ Functionalities of the gut microbiota include modulation of the gut barrier integrity and digestion of indigestible fibers, thereby allowing energy harvest from otherwise indigestible food sources.^9^ Bacterial metabolites herein produced, such as short-chain fatty acids (SCFAs), have been found to regulate host energy and substrate metabolism and are a focal point in research investigating microbiome–host interactions.^9^ Bacterial membrane vesicles (bMVs) are increasingly recognized as important players in microbiome-host interactions, in addition to SCFA’s and other gut-derived metabolites such as bile acids, branched-chain amino acids, tryptophan, and indole derivatives.^10,11^ These bMVs are defined as non-reproducing, lipid-bilayer delimited particles released from bacterial cell membranes that can be generated by both Gram-negative and Gram-positive bacteria.^12^ Characterization studies have found that bMVs are thermostable and readily contain bacterial nucleic acids, metabolites, proteins, and endotoxins.^10–13–16^ They typically range between 20 and 400 nm in diameter^12^ and, conceivably, are able to pass the intestinal barrier in health^17^ and disease^18^ conditions. In contrast to secreted bacterial proteins and nucleic acids that are not encapsulated, vesiculated bacterial factors are protected from proteolytic degradation and nuclease activity.^13–15^ The systemic dissemination and concentrated delivery of these bacterial factors to host cells is facilitated by molecules on the vesicle membrane that interact with the host cell membrane and its receptors, to enable delivery of bMV cargo.^19–22^ In the context of MetS and obesity, researchers have reported an interaction between gut-derived bMVs and insulin sensitive tissues (such as AT, the liver, and skeletal muscle) in rodent studies, with consequential changes in signaling from adipocytes to liver and skeletal muscle (Figure 1).^23–25^

In this review, we will focus on the available knowledge regarding the nature and functionalities of these gut-derived bMVs. We provide an overview of bMV biogenesis, and on mechanisms through which gut bMVs could affect host substrate metabolism in AT, skeletal muscle and the liver. We furthermore highlight potential relations between microbial dysbiosis and metabolic disease, mediated by differences in bMV repertoire (content and functionality) that might arise in overweight/obesity and related metabolic diseases.

## Biogenesis of bMVs in the intestinal lumen

Depending on isolation and counting methods used, 10^10 bMVs can easily be purified from their bacterial producer strains per gram of human fecal matter.^11,26^ The formation of bMVs was first observed to occur through blebbing of the outer membrane of Gram-negative bacteria, typically as a result of cell wall recycling and exposure to stress conditions.^27,28^ In addition to these specific bMVs termed outer membrane vesicles (OMVs), Gram-negative bacteria are known to produce outer–inner membrane vesicles and explosive outer-membrane vesicles whose formations are observed during bacterial cell lysis.^12^ Vesicle biogenesis in Gram-positive bacteria, lacking an outer membrane, is less well understood, but some of the mechanisms describing the formation of bMVs herein involve stress-induced fluidity of the plasma membrane and subsequent membrane protrusion/constriction through the peptidoglycan layer.^29^ This process as well as the lysis of Gram-positive bacteria gives rise to inner-membrane or cytoplasmic membrane vesicles.^12^ Lastly, formation of bMVs is also described for Gram-neutral Mycobacteria.^30^ This evolutionary conservation of bacterial vesiculation is justified by the various roles and functionalities attributed to bMVs, mitigating interactions between microbes^10,16^ and between microbe and host.^31^ It is however important to note that different types of bacteria have various types of partially overlapping vesiculation processes in response to environmental stimuli, and that the classification of bacterial vesicle types is subject to change as new vesicle types and vesiculation strategies are discovered.^12^ A deeper fundamental categorization of bMV nomenclature is described elsewhere.^10^

**Figure 2.**
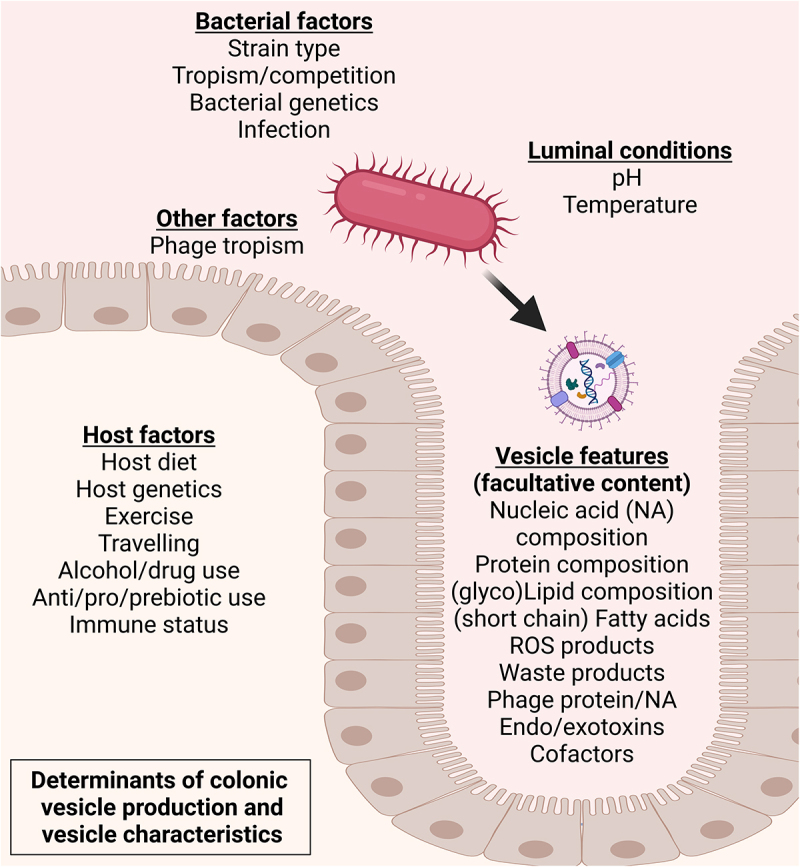
Determinants of bacterial vesiculation and of vesicle characteristics in the large intestine.

Importantly, the factors that could influence bacterial vesicle production and characteristics in the human gut are overlapping those that might influence the bacterial producer strains themselves, and include tropism by bacteriophages,^32,33^ nutrient availability,^23,34,35^ exercise,^36,37^ medication use,^38,39^ and oxidative and/or chemical stress^34^ (Figure 2). However, to date, the potential of altering the gut-derived bMV repertoire to improve human (metabolic) health has not been explored in human clinical interventions. Notably, specific targeting of colonic vesicle production could be attained through deprivation of certain nutrients, given that such measures rapidly induce elaborate stress responses resulting in a change in bacterial vesicle production and characteristics. For example, different bacterial growth stages have been found to produce different vesicle repertoires, as Gram-negative *Bacteroides thetaiotaomicron* produces lipoprotein loaded OMVs at early stages and (lytic) cytoplasmic vesicles at later growth stages.^40^ Authors postulate that such differential vesicle production could be the result of *B. thetaiotaomicron* adapting to nutrient availabilities, and suggest that production of lytic cytoplasmic vesicles is not trivial but a tightly controlled process involved long term bacterial survival.^12,41^ Other examples include the deprivation of iron causing *Vibrio cholerae* to downregulate phospholipid turnover, resulting in increased formation of bMVs in the human host,^42^ as well as cysteine starvation inducing oxidative stress in *Neisseria meningitidis*, yielding increased bMV production in vitro.^43^ In addition to nutrient availability, the exposure to antibiotics has also been found to influence rates of vesicle formation. In Gram-positive bacteria such as *Staphylococcus aureus*,^38^ the exposure to β-lactam antibiotics decreases the integrity of the bacterial peptidoglycan layer leading to increased vesiculation in vitro. For Gram-negative bacteria such as *Pseudomonas aeruginosa*, the exposure to the aminoglycoside antibiotic gentamicin leads to vesiculation by disruption of the cell membrane by interacting with phospholipid moieties.^39^

Given this dynamic nature of vesicle production by the intestinal microbiome, both diet and lifestyle can also impact the nature of bMV (production) by affecting the microbiota themselves. For example, an increased availability of dietary fibers favors the growth of genera such as *Lactobacilli* or *Bifidobacteria*.^44,45^ Simultaneously, this increased availability of dietary fibers has been found to decrease abundancies of colonic mucus-degrading bacteria^46^ and *Clostridium* species.^45^ A decrease in abundance for a certain taxon could occur through competition and selective pressure, both sources of stress. Given that bMV production is described as part of normal bacterial biology as well as a stress response, this suggests that for a given species a decrease in abundance could occur simultaneously with an increase in vesicle production by that species. Such examples illustrate why it is warranted to investigate vesicle repertoires alongside their bacterial producer strains. Additionally, physical exercise has also been found to be a determinant of microbial composition, with most human intervention studies reporting an exercise-associated bloom of acetate and butyrate producing bacteria such as *Bifidobacterium*,^36^ and an increase of benign species such as *Akkermansia muciniphila*.^37^ Again, the effects of diet and lifestyle factors on colonic bacterial vesiculation, and consequential bMV mediated effects on human metabolic health need clarification.

Significant health benefits could be attained when bMV production and properties in the intestine are modulated for health improvement. By for example manipulating agitation rate and lowering pH in in vitro cultures of *Lactobacillus casei* and *Lactobacillus plantarum*, immunomodulatory properties of bMVs released by these bacteria changed as measured by their potential in inducing Interleukin 10 (IL-10) and Tumor Necrosis Factor alpha (TNFα) release by Tohoku Hospital Pediatrics-1 (THP-1) macrophages.^47^ As such, nutritional intervention strategies might benefit from stimulating bacteria to produce bMVs with more benign immunomodulatory properties, for example through inducing a decrease in colonic pH as reported to occur through microbial fermentation.^48,49^ Herein, consumption of indigestible fermentable fibers could be considered as interventional strategy in modulating vesicle production, as well as stimulating SCFA production (extensively reviewed elsewhere^9^). In porcine stool samples a bloom in bMVs as secreted by *Clostridiales*, *Bacilli* and *Enterobacteriales* was observed as a result of dietary administration with the complex fiber β-mannan.^35^ For mice it has previously been found that feeding with a high-fat diet (HFD) results in increased numbers of bMVs in systemic circulation through a decrease in specialized macrophages that clear these vesicles.^50^ Others report an intestinal bloom of Proteobacteria-produced bMVs and concomitant increase in bMV associated lipopolysaccharide (LPS) as a result of a HFD in mice.^23^ Also in mice, a high-protein diet (HPD) was found to significantly increase microbiota vesicle production altogether, when compared to HFD or high-carbohydrate diets.^34^ Such examples illustrate how the intestinal production of bMVs is subject to environmental cues that could be exploited in dietary or lifestyle interventions. Most of such current research however focuses on the effects that specific diets have on the microbiota themselves, with far less focus on bMV biology. A further understanding of molecular pathways governing vesicle formation is crucial, particularly regarding the determinants of their immunomodulatory capabilities and impact on host energy metabolism. Elucidating these determinants holds promise for shaping interventional approaches aimed at modulating bioactive vesicle production in the human host.

Notably, vesicle production rates can vary greatly between bacteria, with a recent study using standard liquid monocultures indicating a bMV/CFU (colony forming unit) count of over 10^5 for *Helicobacter pylori*, and approximately 50 for *Pseudomonas aeruginosa* over the course of 16 h.^51^ A bMV repertoire as produced by the microbiota does therefore not represent the composition and species’ abundancies of the bacterial producer strains with high fidelity, as was recently demonstrated.^11,52^

Herein it is imperative to understand that bMVs are indispensable in microbe-to-microbe interactions, either by for example assisting other microbes in nutrient acquisition, or in the form of mitigating tropism/competition with other bacteria.^10,53^ Exemplifying, these vesicles could facilitate microbial growth through transport of vitamins, cofactors or proteins,^54^ or through encapsulation of hydrolases that facilitate degradation of carbohydrates to be used as nutrients.^55^ Alternatively, microbes have been shown to utilize bMVs for offensive purposes in microbe–microbe communication as well, as bMVs from *Myxococcus* species have for example been found to contain various factors with antimicrobial activity^56^ or some *Escherichia coli* strains produce bMVs that enclose peptidoglycan-degrading enzymes.^19^ Effects on host metabolism could then be a consequence of bMV mediated delivery of bioactive enzymes to host tissues, leading to localized degradation of various substrates. In addition, microbes affecting each other’s growth through secretion of bMVs could influence the abundance of various bacterial taxa. Effects on host metabolism could then be a consequence of a change in composition of the microbiome itself.^7,8^ Nevertheless, bMVs are also a pivotal player in directed interkingdom communication between microbes and the host, and have been found to be well tailored to their role as mediators.^10,28,31^ Exemplifying is the observation that bMVs are equipped with specific adhesion molecules that facilitate the physical interaction with host cells,^19^ and they exhibit various functionalities to escape and modulate the host’s immune system (Table 1). Effects on (human) host metabolic health as a result of delivery of bacterial factors through bMVs require further exploration however, to identify cues for modulation of ‘beneficial’ bMV production.

**Table 1.** Overview of in vivo and in vitro studies investigating involvement of gut-derived bMvs in translocation, (host) inflammation, and/or effects on intestinal barrier function, with potential implications on host metabolic health.

Bacterial origin of bMV	Consequence	Model	Mechanism/process	Detected bMV enclosed/associated factors	Source
**Homeostatic/benign roles for bMVs**					
*Akkermansia municiphila*	Alleviation of adipose inflammation	In vivo (mouse)	*A. municiphila* bMVs reduce the expression of inflammatory cytokines (IL6, TNF TNF-α) in AT and the liver of obese mice	Not described	^24^
*Akkermansia muciniphila*	Enhanced barrier function	Caco-2 cell line	*A. municiphila* bMVs consolidate barrier function through upregulation of Occludin.	Not described	^57^
*Bacteroides fragilis*	Alleviation of intestinal inflammation	In vivo (mouse)	Bacterial release of OMVs associated with capsular polysaccharide PSA stimulates anti-inflammatory cytokine production	Polysaccharide A (PSA), Polysaccharide B (PSB)	^58^
*Bacteroides fragilis*	Alleviation of (intestinal) inflammation	In vitro T-cell and DC coculture and *in vivo* (mouse)	*B. fragilis* OMVs exercise benign immunomodulatory responses through ATGL16L1 signalling in host immune cells, thereby protecting from colitis	Not described	^59^
*Bacteroides fragilis*	Facilitate polysaccharide digestion	In vitro protease assay	Aiding digestion and energy harvest from fiber substrates by encapsulating enzymes in OMVs	Various proteases and glycosidases	^55^
*Escherichia coli* Nissle 1917	Weight loss and normalization of glucose sensitivity	In vivo (mouse)	Exact mechanisms unknown. Observed physiological effects correlated to decreased markers of inflammation and increased SCFA in livers of treated mice.	Outer membrane protein A, outer membrane protein C	^60^
*Escherichia coli* Nissle 1917	Anti-inflammatory cytokine release	Caco-2 cell line	Caco-2 cell model for intestinal barrier exhibited anti-inflammatory signalling in response to *E. coli* Nissle 1917 bMV exposure	LPS	^61^
*Escherichia coli* Nissle 1917 & *Escherichia coli* ECOR63	Enhanced barrier function	*T*-84 and Caco-2 cell line	Cell monolayers reinforced cell-cell adhesion in barrier model through upregulation of tight-junction protein expression in response to bMV exposure	Not described	^62,63^
γ-Proteobacteria *	Potentially dampens pro-inflammatory cytokine release	THP-1 macrophage culture	Increased abundance of γ-Proteobacteria -derived bMVs significantly decreased TNF-α release in THP-1 cells	16S ribosomal DNA, outer membrane protein A, LPS	^11^
**Pathogenic roles for bMVs**					
Actinobacteria*	Pro-inflammatory cytokine release	THP-1 macrophage culture	Increased abundance of Actinobacteria-derived bMVs significantly increased TNF-α release in THP-1 cells	16S ribosomal DNA, outer membrane protein A, LPS	^11^
*Bacteroides fragilis* (BFT+)	Disruption of cell adherence	Human colonic epithelial cell culture	Only bMV-associated BFT causes disruption of epithelial cell-cell contact	*Bacteroides fragilis* BFT	^21^
*Escherichia coli* ECOR12	Pro-inflammatory cytokine release	Caco-2 cell line	Caco-2 cell model for intestinal barrier exhibited pro-inflammatory signalling in response to *E. coli* ECOR12 bMV exposure	LPS	^61^
*Porphyromonas gingivalis*	Insulin resistance in liver	In vivo (mouse)/HepG2 tissue culture	Administration of *P. gingivalis* vesicles diminish insulin-stimulated glycogen synthesis in the liver	Various outer membrane gingipains and periplasmic peptidases	^25^
*Porphyromonas gingivalis*	Pro-inflammatory polarization of macrophages	Murine macrophages and human primary monocytes	*P. gingivalis* derived bMVs induced strong M1 polarization in macrophages	Not described	^64^
*Pseudomonas panacis*	Insulin resistance in skeletal muscle and AT	In vivo (mouse)	Administration of fecal bMVs derived from HFD fed mice, as well as isolated *P. panacis* bMVs, induced insulin resistance in skeletal muscle and AT of lean mice	16S ribosomal DNA, LPS, lipoteichoic acid LTA	^23^
**Conceivably neutral roles for bMVs**					
*Akkermansia municiphila*	Upregulation of PPAR gene expression	Caco-2 and Hep-G2 cell line	*A. municiphila* bMVs increase the transcription of PPAR genes in a dose-dependent fashion	Not described	^65^
*Bacteroides thetaiotaomicron*	Uptake of bMVs by epithelial cells as well as paracellular transport through epithelial layers	In vivo (mouse)/ Caco-2/intestinal organoid	Intestinal epithelial cells take up Bt bMVs through endocytosis followed by co-localisation with the perinuclear membrane, additionally Bt bMVs can pass through epithelial layers resulting in systemic distribution within hours of oral administration	Outer membrane protein A	^17^
*Escherichia coli*	Homing of bMV cargo from intestinal lumen to various host organs	In vivo (mouse)	*E. coli* bMV encapsulated Cre-recombinase enzyme was delivered from intestinal lumen to distal host organs leading to excision of a STOP cassette in host cell DNA and subsequent expression of a reporter gene	Cre-recombinase and green fluorescent protein, both recombinant	^66^

*Abundance of vesicles derived from these bacteria in a heterogeneous bMV mixture was significantly, and in a dose-dependent manner, correlated to a physiological outcome.

Summarizing, research focusing on the health consequences of microbial dysbiosis, in which the metagenomic composition of the microbiome (i.e. relative abundance of gut-residing taxa) is determined in relation to participant characteristics, fails to take into account an aspect of microbial activity and functionality. Given that gut-luminal bMV production reflects a degree of the microbiome’s activity and functionalities, we argue that the studying of these vesicles allows for an increased understanding of (eventual) disease mechanisms. The physiological relevance of studying the astronomical numbers of bMVs in the intestinal lumen is further warranted given that their cargo and membrane associated biomolecules are derived from the bacterial producer under specific conditions, conferring biological, physical, and a plethora of immunological properties.^10^ Again, bMVs can contain bacterial DNA,^13^ (functional) RNA,^14,67^ proteins,^15^ and various cytosolic contents including enzymes, toxins, and bacterial waste products,^16^ yet herein it is imperative to understand that not all bMVs contain (producer-strain specific) biologicals (Figure 2).

## Crossing the intestinal barrier

**Figure 3.**
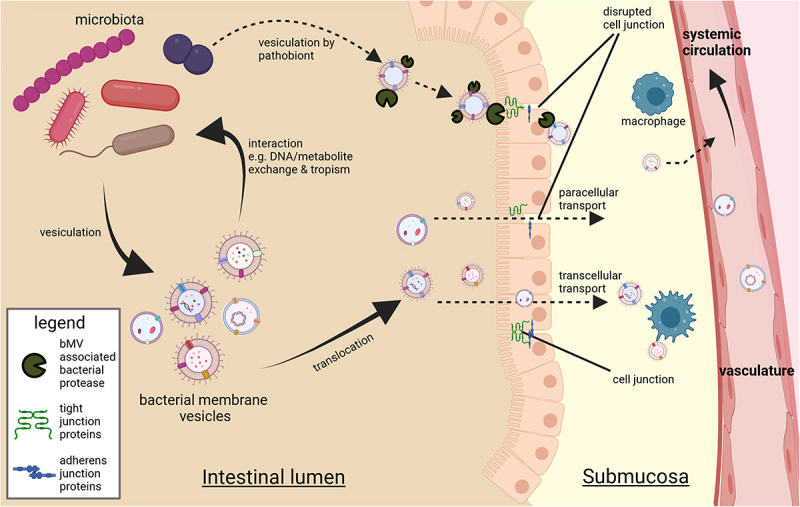
Contextual setting in which the gut microbiota yield a diverse vesicle repertoire interacting with bacterial producer strains and the gut epithelium. Vesicles exiting the intestinal lumen is reported to occur through transcellular migration and through (vesicle induced) disruption of junction proteins regulating intestinal barrier function.^10^ Vesicle-associated bacterial proteases such as *B. fragilis* fragolysins^83,84^ or possibly *P. gingivalis* gingipains^85^ have been reported able to disrupt barrier function leading to increased leakage of colonic contents. In the intestinal submucosa the (increased) presence with bMVs could induce polarization of extraintestinal immune cells^64^ whereas translocation to vasculature facilitates transport of bMVs to distal metabolically relevant tissues and organs.

It is noted that various tissues including blood,^68–71^ AT,^50,70,72^ the liver,^73^ and skeletal muscle^74^ from otherwise healthy human individuals contain bacterial DNA. One group recently reported the occurrence of live bacteria in AT of subjects with T2D and/or obesity using fluorescent in-situ hybridization techniques.^70^ Considering the isolation and detection of bacterial DNA from biological samples, commonly used nucleic acid isolation and purification methods employ chaotropic lysis reagents to release DNA and RNA from complex sample mixtures.^50,68–72^ Such procedures do not allow distinguishing between previously vesiculated DNA, DNA within intact bacteria and “loose” free-floating DNA. In such mentioned studies, the detection of extraintestinal bacterial DNA could potentially be attributed to vesicle translocation from the intestine either via transcellular migration or through paracellular leakage of colonic contents (Figure 3). This in vivo translocation of bacterial vesicles from the intestinal lumen to distant host organs was clearly demonstrated in a seminal mouse study, employing mice with a fluorescent reporter background colonized with Cre-recombinase expressing *E. coli*.^66^ The delivery of bacterially expressed recombinase enzyme to host tissues was herein mediated by bMVs. Delivery of the enzyme to host cells resulted in excision of a stop-cassette upstream of the reporter gene, yielding induced expression in at least the brain, liver, heart, kidney and spleen.^66^ Importantly, considerable knowledge gaps exist in the understanding of gut-borne bMVs, spanning from their point of origin to their mediation of effects on host tissues. A concise overview of this interkingdom communication (encompassing bMV production, translocation, distribution, host-interactions, and elimination) and persisting knowledge gaps has been summarized previously.^31^

In human studies, much research on this topic focuses on extraintestinal bacterial DNA or ‘signatures’ rather than bMVs. Interventional studies on microbiota and metabolic health report for example the presence of bacterial DNA in muscle biopsies and blood of healthy male participants, prior to and following 6 weeks of endurance training.^74^ The authors report a differential abundance of DNA from certain taxa in muscle tissue, with a bloom in for example *Staphylococcus* and *Acetinobacter* signatures post exercise, and suggest underlying changes in gut barrier permeability are involved. Arguably the detection of bacterial DNA in host tissues outside the gastrointestinal tract could involve bMVs, as circulating cell-free DNA (cfDNA) is subject to nuclease activity in blood plasma.^75^ Herein, it is noted that for microbial cfDNA a biological half-life of 16 min to 2 h has previously been reported.^76^ Encompassment within a bMV would shield this DNA from nuclease degradation in circulation. A plausible explanation for an *increased* extraintestinal presence of bacterial signatures such as cfDNA and bMVs is the compromised intestinal barrier function as observed in a number of conditions including obesity,^77^ inflammatory bowel disease (IBD),^78^ HIV,^79^ and older age.^80^ Microbial dysbiosis is typically concomitant with this intestinal barrier dysfunction, in which paracellular transport of luminal components to systemic circulation is strongly increased.^72^ Indeed, increased amounts of bMV associated LPS were detected in plasma samples of IBD and HIV patients when compared to healthy controls, together with increased systemic concentrations of zonulin; a protein believed to be involved in tight-junction maintenance.^81^ Additionally, alcohol consumption has been found to increase the translocation of intestinal and bacterial components such as LPS, from an otherwise healthy human gut into to systemic circulation.^82^ Disease mechanisms and immunological responses in host tissues because of such bMV translocation, enabling transport of bacterial components and metabolites to systemic circulation and peripheral tissues, have been identified in a number of in vivo murine and in vitro studies discussed in this review (Figure 1, Table 1).

bMVs themselves have also been found able to directly influence intestinal barrier function; the interaction between a Caco-2 cell model for the intestinal epithelium and *E. coli* bMVs indicated vesicle uptake by Caco-2 cells resulting in a change in inflammatory signaling in these epithelial cells.^61^ The bMV-associated metalloproteinase fragolysin (a.k.a. *Bacteroides fragilis* toxin or BFT), a prominent virulence factor produced by *B. fragilis*, can disrupt the murine intestinal barrier in vivo through cleavage of E-cadherins that regulate adherence between enterocytes^83,84^ (Figure 3). Interestingly, fragolysin causes epithelial cell contact disruption exclusively when associated to a vesicle membrane.^21^ Similar to this, the presence of intestinal *Porphyromonas gingivalis* in mice has been found to exacerbate gastrointestinal inflammation by disruption of the intestinal barrier function, which is believed to be the result of gingipain (a bacterial cysteine protease) mediated degradation of tight-junction proteins.^85^ Although this work did not focus on the contributing role of secreted bMVs in this pathogenesis, considerable bMV production by *P. gingivalis*^86^ and vesicle association of gingipain protein^87^ are reported in literature, delineating a potentially clinically relevant mechanism through which bMVs could affect the gut barrier function and host health. Where earlier paradigms considered the presence of bMVs in extraintestinal tissues solely as the consequence of a compromised intestinal barrier, more recent findings indicate that bMVs can also pass through an uncompromised intestinal barrier to readily reach peripheral organs such as the liver, lungs, and heart of healthy C57BL/6 mice.^17^ Consolidation of epithelial cell junctions by bMVs has also been reported, with vesicles produced by *E. coli* strains Nissle 1917 (EcN) and commensal ECOR63 reported to upregulate expression of tight-junction proteins in vitro.^62^ The same group described how these ‘benign’ bMVs can counteract intestinal barrier dysfunction caused by enteropathogenic *E. coli* (EPEC).^63^ Similar benign effects are described for *A. muciniphila* bMVs, able to counteract increases in Caco-2 monolayer permeability as a result of exposure to LPS, whilst in this study no effects where observed for *E. coli* bMVs.^57^ In mice, the commensal *B. fragilis* has been reported to produce bMVs associated with polysaccharide A, and these vesicles stimulated anti-inflammatory cytokine production in the intestinal lumen.^58^ Additionally, immunomodulatory molecules secreted through *B. fragilis* bMVs protected against experimentally induced colitis in a similar mouse model.^59^ The favorable properties of these vesicles were mitigated through ATG16L1 and NOD2 signaling in autophagy by host’s immune cells, and authors illustrate how knockout of ATG16L1 abolishes the OMV’s protective effects against colitis. This exemplifies how the interaction between bMVs and specific host genes can contribute to intestinal barrier regulation. Others report *B. fragilis* bMVs to be involved in maintaining gut homeostasis by encapsulating enzymes facilitating fiber breakdown, promoting digestion and microbial energy harvest from complex carbohydrates.^55^ In contrast, the aforementioned fragolysin-associated *B. fragilis* bMVs are reported as harmful.^21^ This illustrates that vesicles produced by a given species could have benign or pathogenic effects on the host, depending on context and associated PAMP repertoire (Table 1). In studying bioactivity of bMVs on host cells, it is noted that the enclosed or associated factors of these bMVs are not always reported or investigated in great detail (Table 1). Arguably, a more holistic approach to assessing bMVs involves consideration of their entire antigenic repertoire. For instance, researchers focusing on bMV associated LPS in *E. coli* report immunomodulatory effects for one strain and pro-inflammatory effects for another.^61^ Detection or quantification of bMV-associated LPS alone herein does not capture the essence of the bioactive effects of bMVs. A more comprehensive understanding of vesicle immunogenicity therefore requires an understanding of all relevant associated molecules, either as presented on the vesicle surface such as receptors or LPS, or as enclosed factors that might be delivered into host cells. In addition to such intrinsic bMV factors, the host microenvironment in which these vesicles interact with host cells also plays a pivotal role, as (circulating) immune cells exhibit heightened sensitivity to pathogen-associated molecular pattern (PAMP) cues compared to other somatic cell types.^50,88^

Regarding biodistribution studies of bMVs, in which gut-derived bMVs are detected in extraintestinal niches, most researchers still rely on animal models. Notably, the bMVs’ distribution and ability to cross barriers is for example a focal point in research investigating the gut-brain axis. Oral administration of bMVs from *Paenalcaligenes hominis* to murine models for Alzheimer’s disease (AD) resulted in a significant increase in detected bacterial 16S ribosomal DNA in brain tissues, concomitant with local inflammation, and cognitive decline.^89^ In the context of metabolic disease, Choi et al. reported orally administered vesicles from *Pseudomonas panacis* to induce insulin resistance and impaired glucose metabolism in mice.^23^

In humans, postmortem analysis of brain tissue of AD patients and controls readily indicated the presence of DNA originating from Actinobacteria, Firmicutes, Proteobacteria and *Bacteroides*.^90^ However once more the use of chaotropic lysis reagents used in this work, to release nucleic acids from tissue samples by dissolving DNA-enveloping structures, disenables the detection of bMVs. In such research it is noted that bacterial DNA could have been contained within whole bacteria, phagocytosing immune cells, protein (aggregates), or bMVs. Generally, authors however do not elaborate on the exact nature of the biological envelope of this DNA, shielding it from nuclease degradation, but postulate that an aging-related decrease in the integrity of endothelial barriers could contribute to translocation of bacteria and their products.^90,91^ A process involving translocation of gut-derived bMVs through the blood–brain barrier might herein also help explain the detected presence of bacterial LPS and *E. coli* pili proteins in the older healthy human brain.^92^ Interestingly, a diverse gut-derived bMV repertoire was recently characterized in human breast milk,^93^ suggesting that bMVs could potentially translocate from the gut to the mammary glands. Ongoing advances in the development of sensitive bMV separation and detection methods using complex matrices (e.g. blood, milk, or liquor) are herein essential to understand the impact of circulating gut-borne bMVs.^31^ To date, however, clinical human evidence linking translocation of gut-derived bMVs (as opposed to bacterial DNA or ‘signatures’) to the etiology of (metabolic) disease, is lacking.

## bMVs and insulin sensitive tissues

### bMVs in AT

**Figure 4.**
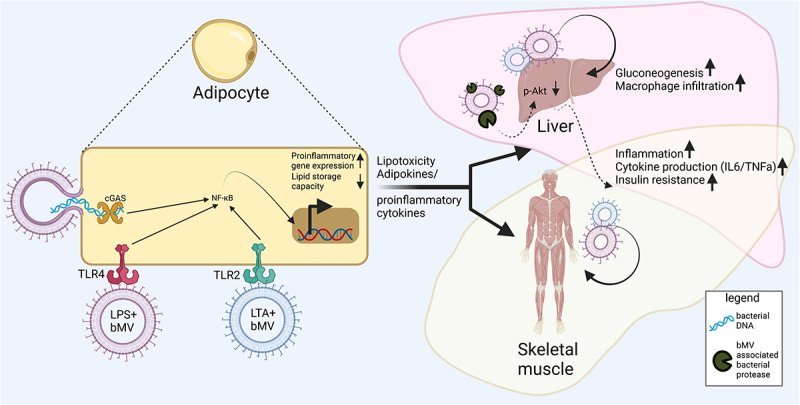
Interactions between bMVs and metabolic tissues. Reported consequences implicated in metabolic disease mostly involve proinflammatory signalling in AT, the liver and skeletal muscle. Mechanisms through which bMVs mediate their biological effects are via activation of pathogen recognition receptors (PPR) such as Toll-like receptors TLR4^100^ & TLR2^101^ and cyclic GMP-AMP Synthase (cGAS).^95^ In addition, bMV associated proteases can dysregulate glucose homeostasis through inhibition of insulin signalling in the liver,^25^ via mechanisms not fully understood. Upregulated expression of pro-inflammatory genes following PRR activation occurs through translocation of nuclear factor kappa-light-chain-enhancer of activated B cells (NF-kB) to the cell nucleus.^107^ Proinflammatory signalling in AT is concomitant to a decreased AT lipid storage capacity. A subsequent lipid overflow to non-AT tissues acts in concert with proinflammatory endocrine AT signalling to the liver and skeletal muscle.^6^

The consequences of gut-derived bMVs homing to metabolic tissues, particularly AT, have been studied in obese mouse models.^50,94^ In these models, the translocation of gut-derived bMVs is associated with AT inflammation and tissue-specific insulin resistance.^50^ This finding was substantiated by incubation of human microbiome derived bMVs with human and mice AT and liver cells in vitro.^50^ The effects of bMV-induced inflammation were exacerbated in CRIg+ (specialized macrophages clearing bMVs from circulation) knockout mice and diminished upon depletion of bMV DNA,^50^ suggesting that the delivery of bacterial DNA is at least in part responsible for bMV-induced inflammatory effects on the host^95^ (Figure 4). In obese mouse models, the CRIg+ macrophage population has been found significantly reduced, resulting in a strong increase of bMV translocation to AT.^50^ The subsequent enrichment of gut-derived bMVs in AT, as detected by a marked increase in bacterial DNA, is accompanied by local inflammation and aggravated insulin resistance.^50^ In human subjects, however, a clinically significant influence of bMV capture by CRIg+ macrophages, on parameters such as AT inflammation has not been demonstrated yet. Another important notion is that the mesenteric adipose tissue (mAT) surrounding the large and small intestine is increasingly recognized in acting as a second intestinal barrier, through sequestration of bacterial signatures originating from the intestinal lumen.^96,97^ In line with this idea is that in mice the removal of mAT resulted in hepatic inflammation and reduced overall glucose tolerance concomitant with an increase in bacterial load (DNA) in the liver,^98^ suggesting a causal link between translocation of intestinal contents and metabolic disease.

In humans, more recent work on visceral AT depots sampled during bariatric surgery of 40 patients with severe obesity (average BMI 50.5 kg/m^2^), a considerable load of bacterial DNA (average >10.000 copies of bacterial 16S rRNA/mg of tissue) was detected in omental, mesenteric, and subcutaneous AT whilst not in plasma (average <100 copies of bacterial 16S rRNA/mL).^99^ The authors herein also investigated these extraintestinal bacterial signatures in T2D and found striking differences in bacterial signatures in mAT dictated by glucose sensitivity, independent of BMI. Interestingly, when comparing all sampledATs the highest average number of operational taxonomical units was detected in mAT, further illustrating its role in sequestration of bacterial signatures surpassing the ‘first’ intestinal barrier.^99^ Such work provides evidence for selective and tissue specific microbial signatures in obesity, potentially identifying new therapeutic targets and/or biomarkers in metabolic disease. The presence of bMVs (as envelope of this bacterial DNA) was however not investigated in this work of Anhê et al.,^99^ and given the low concentrations of bacterial DNA in bMVs^11^ it is important to take along stringent and elaborate controls to recognize detection of contaminating bacterial DNA from the environment, resultant from laborious sample collection and processing.^72^

Thus far, adipocyte dysfunction as a result of gut-derived bMV exposure has been largely explained through vesicle mediated inflammation by transport of TLR4^100^ and TLR2^101^ ligands and bacterial DNA^50^ (Figure 4). Subcutaneous administration of peptidoglycan, a prominent TLR2 ligand as found on Gram-positive bacteria as well as their vesicles,^10^ increased serum triglyceride concentrations in mice^102^ which was concomitant with macrophage infiltration and inflammation of AT.^103^ Similarly, for the Gram-positive endotoxin lipotechoic acid (LTA), also present on bMVs,^12^ a dose-dependent increase in serum triglycerides and carbohydrate oxidation was observed following intraperitoneal administration in rats.^104^ AT infiltration by macrophages could herein also be the result of bMV-induced immune signaling through cGAS-STING in these macrophages, where the exposure of these macrophages to vesicle-enclosed bacterial DNA is responsible for pro-inflammatory responses.^95^ In human plasma, the presence of bMV-associated LPS was found upregulated in IBD patients, HIV patients, and people undergoing radiation- or chemotherapy, and this upregulation was found to be concomitant with intestinal barrier dysfunction.^81^ Human peripheral blood mononuclear cell activation by such bMV-associated LPS was also reported, although the authors did however not focus on eventual immune infiltration of AT in this setting.^81^ In the context of host energy and substrate metabolism, there is currently still a need for studies investigating what the consequences of association of a microbiome-derived bMV with specific PAMPs are.

Nevertheless, for both Gram-positive and Gram-negative vesicles, many described pathophysiological effects on host cells are likely mediated through recognition of bMV-associated PAMPs. Binding of PAMPs to a host receptor herein results in translocation of pro-inflammatory transcription factors to the host cell nucleus to yield a pro-inflammatory response (Figure 4). Properties of bMVs can therefore be characterized by means of analyzing their associated and enclosed PAMPs. Although not all aforementioned studies that investigate PAMP signaling focus on bMV association, it is emphasized that co-administration of antigens with bMVs could boost stability and immunogenicity of an antigen.^22^ Investigations into specific homing of gut-derived bMVs to certain target tissues in vivo, dictated by the PAMP repertoire of these vesicles, are currently lacking but could facilitate improved understanding of these microbiome-host interactions.

Considering MetS, in which related features of diabetes and obesity co-occur, AT plays a central role in the interplay between metabolic organs. Considering the often observed enrichment of gut-derived bMVs and other bacterial signatures in dysfunctional and inflamed AT,^50,70,99^ we postulate that these bMVs merit attention in studying organs with dysfunctional energy/substrate metabolism in metabolic disease (Figure 4). For example, white adipocytes are largely responsible for the removal of triacylglycerol from systemic circulation, sequestering them for energy storage, and allowing release of this energy through the process of lipolysis in which free fatty acids are released.^105^ When (white) adipocytes’ lipid storage capacity is exceeded or compromised as a result of disease, lifestyle, genetics,r inflammation (e.g. by gut-derived bMVs and/or subsequent macrophage infiltration), excess lipids will enter systemic circulation and accumulate in non-ATs.^88^ This ectopic fat accumulation in other insulin sensitive tissues such as skeletal muscle and the liver, can induce lipotoxicity, a condition in which excess lipids and accumulated bioactive lipid metabolites in non-AT impair endogenous cellular functions, resulting in impaired insulin signaling in skeletal muscle and the liver.^88^ The development of insulin resistance is a main precursor for T2D, and commonly observed in obesity.^5^ Yet, the nuances affecting the interplay between various tissues in the pathogenesis of insulin resistance, are still under investigation. Mounting evidence suggests that he (gut-)microbe-host interactions play a critical role.^4^ We postulate that bMVs are a potentially critical player in the etiology of metabolic disease, given their production by the microbiome and ability to induce localized inflammation and changes in substrate metabolism in the most relevant metabolic tissues. This is further exemplified by the observation that intestinal barrier (dys)function itself influences the crosstalk between AT, skeletal muscle and liver cells in obesity and T2D.^106^ In metabolic endotoxemia, defined as a condition in which two to threefold increased levels of LPS enter the systemic circulation due to compromised intestinal barrier integrity, increased levels of pro-inflammatory cytokines (e.g. IL6 and TNFα) are detected in visceral and subcutaneous AT, muscle, and liver tissues.^106^ Again, a compromised intestinal barrier has also been linked to the presence of bMV-associated LPS in systemic circulation.^81^ This illustrates the urgent need for human investigations looking into bMV infiltration of these tissues in metabolic disease. Nevertheless, in the previously discussed work^106^ no emphasis was placed on the potential role that gut-derived bMVs could have played in the intestinal barrier dysfunction, or the subsequent local production of pro-inflammatory cytokines by metabolic tissues.

To summarize, translocation of bMVs from intestinal lumen to AT would be concomitant to translocation of non-bMV bacterial products such as LPS, and involved in the onset and progression of metabolic disease as host tissues can exhibit inflammatory responses in the presence of these biologicals. This is evidenced by the observation of in vivo sequestration of bacterial signatures in various AT depots and systemic circulation. The specific consequences of this sequestration might be tissue specific and most likely determined by the concentration and nature of these biologicals (bMV type, bMV with or without PAMPs, PAMP type, etc.), and secondarily through resultant interorgan crosstalk complicating the study of direct mechanistic effects. However, in vivo studies with a primary focus on the effects of gut-derived bMVs on human AT function are necessary to fully understand the role of these vesicles in the pathophysiology of metabolic disease.

### bMVs in the liver and skeletal muscle tissue

There is a lack of human studies examining the consequences of translocation of various bMVs and vesicle-associated PAMPs from the intestinal lumen, on metabolic tissues like the liver and skeletal muscle. It is noted that the nature and amount of such vesicle-associated PAMPs are strong determinants of the effects these bMVs would have on host tissue function. For example, bacterial peptidoglycan and LTA by themselves are potent activators of innate immune receptors^107^ and subcutaneous administration of peptidoglycan has been found to induce liver fibrosis and inflammation in mice.^102^ Upon intraperitoneal LTA administration in rats, elevated levels of serum triglycerides have been detected as a result of increased hepatic secretion.^104^ Although peptidoglycan, LTA and LPS can all be detected on gut-derived bMVs,^10^ such work is often performed with non-bMV associated PAMPs. Hence, given that bMV-association could boost immunomodulatory potential of such antigens,^22^ we advocate that investigations looking into the role of vesicles in these host-microbe interactions are urgently needed.

Exemplifying PAMP-bMV functionality; the aforementioned *P. gingivalis* associated proteases such as gingipains, can exploit the vesicle’s ability to readily translocate into systemic circulation, and their translocation to the liver has been found to induce local insulin resistance in mice, as demonstrated by a decrease in protein kinase B (Akt) phosphorylation^25^ (Figure 4). This example illustrates how a single bacterial protease has tissue-specific pathophysiological consequences as a result of being vesicle-associated. Nevertheless, not many such examples are known given the heterogeneity of gut bacteria and their metabolites. With respect to a species-specific bMV responsible for a disease phenotype, an elegant study conducted by Choi et al. identified *P. panacis* bMVs from a heterogeneous stool bMV population as able to inhibit insulin signaling in murine skeletal muscle cells as well as in AT.^23^ The authors further report the presence of *P. panacis* bMVs, but not bacteria, in skeletal muscle tissue 12-h post oral administration. Strikingly, this infiltration was concomitant to typical diabetic phenotypes as signified by glucose intolerance after glucose administration or insulin injection, suggesting the homing of a gut-derived bMV to host organs might hold much clinical relevance in the context of metabolic disease.^23^

It is complicated, however, to discern direct effects of specific bMVs on specific-host tissues from the effects mediated through pro-inflammatory signaling through immune cells, cytokines or adipokines. In the study of bMVs in vivo, often involving oral^23–25,58,60,66^ administration of bMVs, the main metabolic tissues such as the liver, skeletal muscle, and AT are affected simultaneously. Shi et al. for example studied the effects of orally administered *E. coli* Nissle 1917 bMVs on microbiome composition and liver metabolism in mice.^60^ It was found that two weeks of treatment with these bMVs modulated microbiome composition and reduced body weight in obese and T2D mice, and authors suggest this might be related to increased SCFA concentrations measured in the livers of bMV treated mice.^60^ The underlying molecular mechanisms however remain unknown, and no focus was put on biodistribution of these *E. coli* bMVs to the liver or other metabolically relevant tissues. Studying tissue homing of bMVs as performed by Bittel et al.^66^ would herein further the understanding of effects that these vesicles might have on host tissues, either through or in concert with SCFAs. Nevertheless, studying direct effects of bMVs on the liver, skeletal muscle, and other tissues might benefit from an in vitro setup that does not take into account confounding signaling between AT, liver and skeletal muscle.

In humans, previous work on a cohort of liver cirrhosis patients highlighted that disease severity, defined in terms of intrahepatic endothelial dysfunction, exhibited a positive correlation with the presence of gut-derived bacterial DNA in plasma.^108^ Again, however, here no distinction was made between vesiculated and ‘free’ DNA, suggesting that perhaps the translocation of bacterial DNA here is in the form of bMV translocation concomitant with various PAMPs. As such, effects of bMVs on skeletal muscle and liver could readily occur through inflammation mediated by adipokine signaling (Figure 4) or by macrophage polarization,^11^ resulting in tissue infiltration and insulin resistance.^50^

Evidence for direct mechanistic effects of bMVs on host substrate metabolism other than through inflammation is scarce, but recent work on *A. muciniphila* and its derived bMVs indicated that these vesicles might directly increase transcription of peroxisome proliferator-activated receptor (PPAR) genes in HepG-2 (hepatocyte-derived) and Caco-2 cells.^65^ PPAR genes are members of a family of nuclear receptors best known for their involvement in host glucose and lipid metabolism.^109^ The reported dose dependent effects in increasing expression of PPAR genes at the transcriptional level in Caco-2 and Hep-G2 cells, suggests that *A. muciniphila* bMVs could aid in prevention of obesity and metabolic disease.^65^ The authors furthermore note that *A. muciniphila* readily produces SCFAs which are also known to activate expression of PPARα (promoting fatty acid oxidation), and indicate that the bacteria itself, either heat-inactivated and in active form, as well as cell-free bacterial supernatant, can induce increased expression of these genes.^65^ However, as no focus was placed on the effect of treatment on expression of (anti-)inflammatory genes, it is hard to discern if observed results on PPAR genes are due to direct signaling through bMV enveloped PPAR ligands or (in part) by a change in the cell’s inflammatory state by bMV PAMPs. In addition it would be interesting to investigate whether the SCFA uptake by host cells is promoted by bMVs, for example through SCFA encapsulation and subsequent delivery through membrane fusion. The use of lab-grown bacterial (mono)cultures in the study could herein however have yielded bMVs and metabolites different in nature or concentration than those found in vivo. Nonetheless, increased hepatic PPAR expression might protect against liver disease in obesity and T2D,^110^ but current findings on *A. muciniphila* bMVs remain to be confirmed in vivo and on expression level.

Summarizing, there currently is no good understanding of how gut-derived bMV signaling affects substrate metabolism in skeletal muscle and the liver in humans, and what the implications of such signaling on whole body energy metabolism would be in the context of obesity and T2D. However, the association of gut-derived bMVs with PAMPs or vesicle encapsulation of bacterial metabolites could mitigate significant effects on host metabolism following translocation to these tissues.

### Therapeutic modulation of bMV production in vivo

Vesicle production by the intestinal microbiome is subject to external cues, and as such this yields possibilities for therapeutic modulation to steer the microbiota vesicle production toward producing bMVs with benign properties. Various factors could influence bacterial vesicle production and characteristics in the human gut (Figure 2), and of particular relevance in the context of obesity and T2D are modulation through lifestyle interventions. In humans, nutritional intervention^4^ as well as exercise^36^ have been found to affect bacterial composition, but studies focusing on vesicle production and therapeutic modulation hereof are currently lacking. In vitro characterization of factors and conditions that affect vesiculation might provide essential insights in ways to modulate microbiome bMV production in vivo. Exemplifying; dietary fiber supplementation could promote microbial fermentation to induce a decrease in colonic pH, and changes in overall microbial compositions.^48,49^ In vitro, such a decrease in ambient pH is reported to induce LPS modifications in *Salmonella enterica*, followed by the release of these modified LPS through vesiculation.^111^ Studying examples of such modulation of bMV production by specific gut microbes is complex however, given the heterogeneity and dynamic nature of the microbiome. The effects of physical exercise interventions on gut-derived bMV repertoire are currently unexplored, but exercise-associated blooms of specific taxa such as *Bifidobacterium* have previously been reported in humans.^36,37^

Animal studies have provided some insights in effects of nutritional supplementation on colonic bMV production; complex fiber has been found to increase bMV production by certain taxa (*Clostridiales, Bacilli*, and *Enterobacteriales*) in pigs.^35^ In mice, HFD increased overall bMV production by Proteobacteria^23^ whilst simultaneously decreasing systemic bMV clearance by macrophages.^50^ Also in mice, a HPD increased microbiota vesicle production altogether.^34^ It is hereby noted that in the characterization of vesicles by means of identifying their bacterial producer strains (e.g. by sequencing bacterial DNA/RNA), differences at the lowest taxonomical level could still be relevant. Exemplifying; within species even strain differences can be determinants of vesicle properties, as different *Staphylococcus aureus* strains cultured under identical conditions produce vesicles that vary in the amount to peptidoglycan they carry.^107^ In line with this is that in vitro characterized bMVs derived from *E. coli* and *B. fragilis* have been reported as benign by some and pathogenic by others (Table 1).

Investigating possibilities for lifestyle-modulation of gut-derived bMVs repertoire would be of much interest to researchers studying microbiome–host interactions, yet clinical human evidence for amelioration of host metabolism mediated through gut-derived bMVs is currently lacking.

## Concluding remarks

Addressing the global obesity pandemic with its associated conditions such as T2D requires a holistic understanding of the disease pathophysiology. It is now understood that the gut microbiome plays a critical role in maintaining (metabolic) health, given that various diseases can be exacerbated by – or be the result of microbial dysbiosis. In addition to microbiome derived SCFA’s, bMVs have gained attention because of their rigid nature in combination with the ability to transport bacterial molecules to host organs. Previous work on functional bMV in-vivo mouse studies employed administration of purified vesicles originating from a donor^23,89^ or from monoculture,^17,24,25,66^ indicating context-specific effects on host inflammatory status and/or glucose sensitivity. In vitro work readily allows studying of tissue-specific molecular mechanisms of bMV signaling (Table 1), but lacks a degree of complexity that is the result of signaling between the prime insulin sensitive organs AT, liver and skeletal muscle. Additionally, both bMV administration in vivo^17,23,24,25,66,89^ as well as incubation in vitro^11,21,57,58,61,62,63,64,65^ might employ localized doses of bMVs that would probably not occur under normal physiological conditions. Investigating extraintestinal gut-derived bMV repertoires under normal physiological conditions is difficult however, notably by experimental complications in purifying and characterizing bMVs from these tissues. For example, sensitive techniques employing PCR-based amplification of bacterial nucleic acids can specifically detect small amounts of vesicle-enclosed DNA, but it is possible that bMVs carrying antigens do not contain genetic material. As such it is paramount to take along stringent controls when sampling host tissues,^72^ since contaminating environmental bacteria contain much more DNA and PAMPs than vesicles alone.

Particularly in human studies, the effects of extraintestinal gut-derived bMVs on host metabolism need much further elucidation, but an understanding of this intricate aspect of microbiota-host interaction could pave the way for therapeutic modulation of the microbiota and their vesicle production to ameliorate metabolic health. We argue that gut-bacterial signatures detected outside of the gastrointestinal tract in healthy human subjects could well be the result of bMV translocation, whilst numerous examples already illustrate how microbiota-derived vesicles could exert benign and pathogenic effects on the host. The fact that gut-derived bMV repertoires can be subject to external cues dictating aspects such as physiochemical properties and cargo, explains how they can constitute significant additional complexity in microbiome-host interaction,^26,52^ adding to the complexity of the bacterial communities that produced them. A better perspective on metabolic health thus requires an understanding of the distinction between the effects of intestinal microbes, microbiota-derived metabolites, and bMVs on host metabolism. Except for a few defined molecular mechanisms however, ways which in which gut residing bacterial species, genera, or co-abundance groups can contribute to metabolic disease via bMVs in vivo are still largely unknown.

In conclusion, we hypothesize that the role of bMVs in microbiota–host communication is much more prominent than previously thought. Even though there is currently only limited clinical human evidence for gut-derived bMVs as drivers of metabolic disease, exploring their impact on host tissues (notably through pro-inflammatory signaling) holds great promise for expanding our comprehension of the microbiome-host interplay. As such, the characterization of these vesicles, their biogenesis, and their properties could pave the way for novel therapeutic interventions in the context of obesity and related co-morbidities such as T2D.

## Key messages

**What is already known about this subject?**

- The interkingdom communication between gut bacteria and their human host is mediated by bacterial products (including e.g. gut-derived hormones and metabolites), able to exit the intestinal lumen to reach systemic circulation. The production of such bacteria derived molecules can be modulated by nutrition and lifestyle.
- bMVs are nanosized membrane-delimited particles produced in large amounts by most bacteria, containing bacterial cell constituents, metabolites, and toxins alike.

**What are new findings in this field and how could they impact future research and clinical practice?**

- Rigid, thermostable, and bioactive bMVs can be isolated from fecal matter to shed light on what the bacteria are producing. Future research on the nature and functionalities of these vesicles could help explain metabolic disease phenotypes.
- The bacterial composition of the gut-microbiome is generally different than the bacteria-derived vesicle composition, suggesting characterization of baseline microbial composition does only partly explain metabolic disease phenotype.
- A holistic understanding of microbiome-host interactions taking into account all players paves the way for more personalized interventions to treat and/or prevent metabolic disease.
